# New permanent bundle-branch block and long-term prognosis of patients with new onset ST-elevation myocardial infarction who underwent percutaneous coronary intervention

**DOI:** 10.3389/fphys.2022.892623

**Published:** 2022-08-22

**Authors:** Yi Yang, Jun Wang, Bing Wu, Yanan Xu, Long Tang, Haibing Jiang, Benfang Wang, Tongjian Zhu

**Affiliations:** ^1^ Department of Cardiology Fourth Ward, The Xinjiang Medical University Affiliated Hospital of Traditional Chinese Medicine, Urumqi, China; ^2^ Department of Cardiology, Affiliated Hospital of Xinjiang Medical University, Urumqi, China; ^3^ Department of Cardiology, Urumqi, China; ^4^ Respiratory Medicine, The People’s Hospital of Xuancheng City, Anhui, China; ^5^ Department of Cardiology, Renmin Hospital, Hubei University of Medicine, Shiyan, China; ^6^ Department of Cardiology, The First Affiliated Hospital of Bengbu Medical College, Bengbu, China; ^7^ Department of Cardiology, Xiangyang Central Hospital, Affiliated Hospital of Hubei University of Arts and Science, Xiangyang, China

**Keywords:** right bundle-branch block, left bundle-branch block, gensini score, cohort study, major adverse cardiac and cerebrovascular events, ST-elevation myocardial infarction

## Abstract

**Aim:** The aim of the study was to evaluate the potential predictive value of permanent RBBB and LBBB for longer-term prognosis in patients with new-onset STEMI who underwent percutaneous coronary intervention (PCI).

**Methods:** Patients with new-onset STEMI that underwent emergency PCI at our department from June 2012 to September 2020 were included in the study. Gensini score (GS) was employed to evaluate the severity of coronary lesions. The primary endpoint of the study was the occurrence of major adverse cardiac and cerebrovascular events (MACCEs), the composite of cardiac mortality, recurrence of myocardial infarction, cardiac shock, stroke, stent thrombosis, or revascularization. We also set all-cause mortality as a secondary endpoint.

**Results:** Out of the 547 patients, 29 patients had new-onset permanent LBBB, 51 patients had new-onset permanent RBBB, and 467 patients had no bundle-branch block (BBB). The occurrence of no BBB, new permanent LBBB, or RBBB was not associated with the severity of coronary artery lesions as evaluated by the GS. After follow-up at an average of 43.93 months, MACCEs occurred in 52 patients. Kaplan-Meier analysis showed that patients with new-onset RBBB were at greater risk for MACCEs compared to those with new onset LBBB (χ^2^ = 5.107, *p* = 0.021). Also, an independent correlation was found between new permanent RBBB and LBBB and MACCEs risk. The adjusted hazard ratios (HRs) were 6.862 [95% confidence interval (CI) of 3.764–12.510] for the new-onset permanent RBBB and 3.395 (95% CI of 1.280–9.005) for LBBB, compared to those with no BBB, respectively (both *p* < 0.05).

**Conclusion:** New onset permanent RBBB in patients with new onset STEMI who underwent PCI may be correlated independently with increased risk of poor long-term prognosis.

## Introduction

ST-elevation myocardial infarction (STEMI) is the most common type of acute coronary syndrome (ACS), which is more likely associated with worse clinical outcomes and prognosis compared to unstable angina (UA) and non-STEMI (Vernon Stephen Coffey D’Souza et al., 2019). Therefore, it has become a consensus that for any STEMI patient who is admitted to the emergency department exhibiting acute chest pain, a 12-lead electrocardiogram (ECG) must be immediately obtained and interpreted within 10 min. Accumulating evidence suggests that ACS patients presenting with a left bundle branch block (LBBB) or right bundle branch block (RBBB) generally carry a high burden of morbidity and increased risk of mortality (Van de Werf et al., 2008; Widimsky et al., 2012). Accordingly, identification of patients presented with bundle branch block (BBB), especially new onset LBBB, remains of clinical significance and an indication for an urgent reperfusion therapy in current cardiovascular practice (RBB, 2022). Recent studies report that the presence of RBBB in patients with ACS is a high-risk ECG feature and a predictor of poor clinical outcomes (Antman Elliott et al., 2004; Widimsky et al., 2012; Chan et al., 2016; Paul et al., 2020). This reinforces the concept that new onset RBBB in coronary artery disease (CAD), even in the absence of ST elevation, is associated with unfavorable prognosis (Widimsky et al., 2012; Chan et al., 2016).

Previous studies have found that the occurrence of RBBB is more frequent than LBBB, because the Purkinje fibers of the right bundle branch are longer and structurally thinner than those of the left bundle branch (Sorensen et al., 2013; Meyer Matthias et al., 2020). This difference may contribute to the high susceptibility of RBBB to myocardial ischemia compared to LBBB in ACS. Of note, previous studies on RBBB correlated with high-risk of clinical characteristics and mortality yielded inconsistent results regarding the prognostic significance of RBBB (Antman Elliott et al., 2004; Widimsky et al., 2012; Chan et al., 2016; Paul et al., 2020). Furthermore, to the best of our knowledge, it remains debatable whether new permanent RBBB should be used as an independent variable for long-term prognosis of new onset STEMI in patients following primary percutaneous coronary intervention (PCI). Thus, we sought to evaluate the significance of new permanent RBBB and LBBB for long-term prognosis in new onset STEMI patients who underwent PCI.

## Methods

### Patient recruitment and study design

**FIGURE 1:**
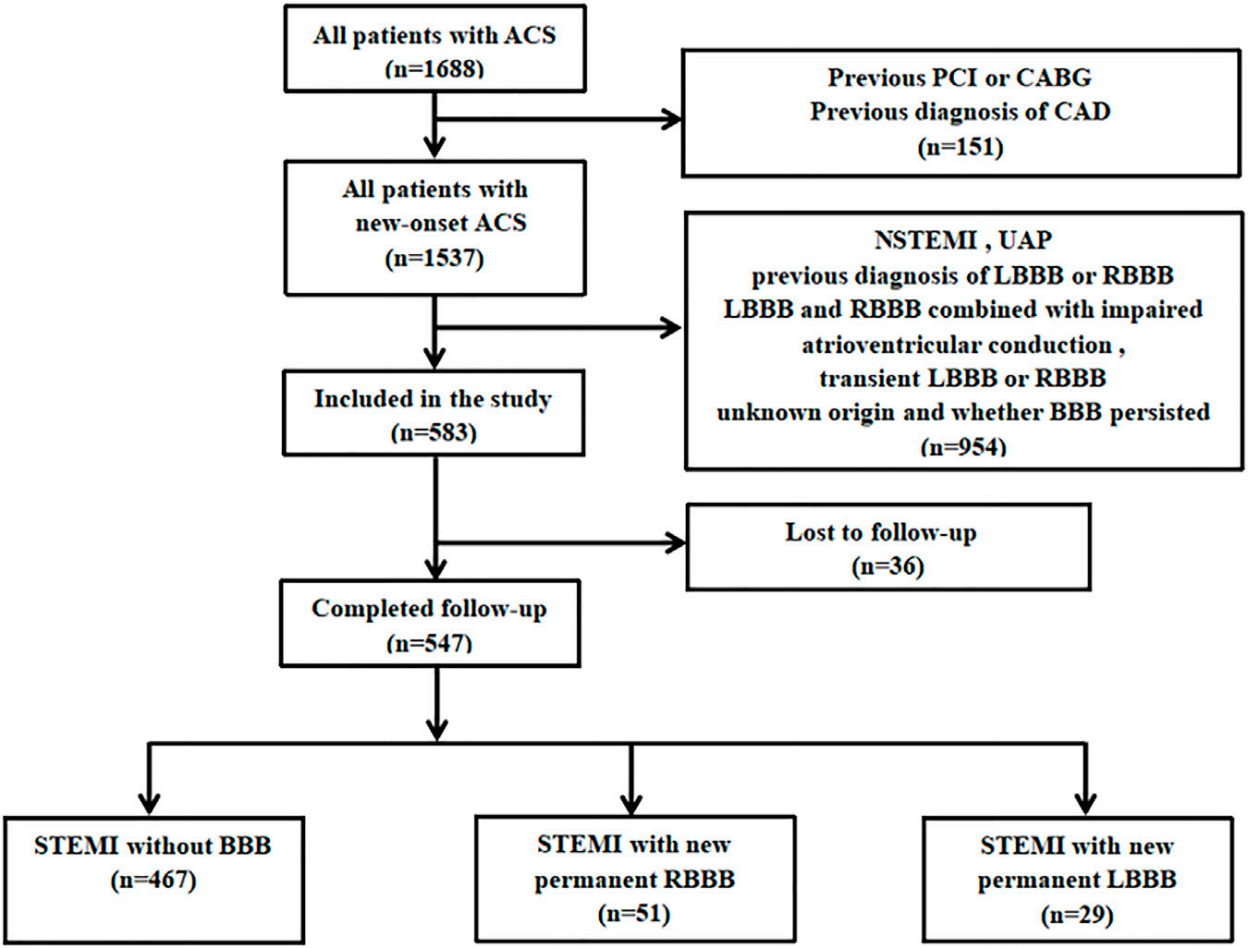
Flowchart of patient enrollment.

Patients with new onset STEMI and without previously known CAD who underwent urgent coronary angiography and PCI from Xinjiang Medical University Affiliated Hospital of Traditional Chinese Medicine from June 2012 to September 2020, were selected for the study. STEMI diagnosis was in accordance with the previously published guidelines (Kawashima and Sasaki, 2011). Patients with any of the following clinical conditions were excluded from the study: 1) previous diagnosis of CAD; 2) diagnosis of non-STEMI or unstable angina pectoris with prior revascularization treatments, including percutaneous coronary intervention (PCI) and coronary artery bypass surgery; 3) previous diagnosis of LBBB or RBBB, transient LBBB or RBBB, new-onset STEMI without PCI, LBBB and RBBB combined with impaired atrioventricular conduction, or left posterior hemiblock and left anterior hemiblock with unknown origin, regardless of the persistence of the BBB; 4) pacemaker implantation; 5) atrial septal defect, aortic stenosis, atrial septal defect, dilated cardiomyopathy, hyperkalemia, digoxin toxicity, rheumatic heart disease, cor pulmonale, ventricular hypertrophy, myocarditis or cardiomyopathy, cardiac conduction system degenerative disease, or primary cardiac fibrosis of the cardiac conduction system; or 6) poor compliance to treatment. Due to the nature of retrospective observational studies, the protocol of this work was approved, and the requirement for the informed consent from eligible patients was waived by the Ethics Committee of the local institution. The flowchart of participant enrollment is shown in Figure 1.

### Blood sampling and electrocardiographic patterns

Peripheral venous blood samples were drawn, and electrocardiography (ECG) was performed immediately upon admission of patients to the emergency department (ED) or right before the urgent coronary angiography. The blood samples were immediately taken for blood cell counts, biochemical analysis of lipids and glucose metabolism, and renal function analysis. Preoperative evaluation included cardiac echocardiography and ECG. All patients were monitored by ECG for seven consecutive days or received a dynamic electrocardiogram after the operation. ECGs were read by an experienced cardiologist at the ED and reviewed once again at the preparation of this manuscript by another independent and seasoned cardiologist. Standard ECG criteria were used to diagnose LBBB and RBBB (Arslan et al., 2018). New onset permanent LBBB and RBBB were considered if they appeared either on admission or before an urgent coronary angiography, but not at baseline.

### Coronary angiography and gensini scoring

After admission, all patients with STEMI were given an emergency coronary angiography and treated with the standard protocols for second generation drug eluting stent placement by experienced and senior cardiologists. Prior to the PCI, loading doses of aspirin and clopidogrel were administered to each patient. The PCI was performed by a group of experienced senior physicians based on the coronary anatomy and clinical conditions of each individual patient. After the PCI, all patients were given the guideline-directed standard treatment regimens, including contemporary antiplatelet therapy and standard-intensity statin therapy, and were followed-up regularly at the clinic after their discharge (Kawashima and Sasaki, 2011). Coronary atherosclerosis was evaluated using the Gensini scoring (GS) system by two experienced cardiologists independently. In case of any disagreements, a third cardiologist would be called in to evaluate the coronary atherosclerosis of the patients, and the final diagnosis was achieved based on the consensus of all three cardiologists.

### Follow-up

The average follow-up period was 43.93 months and was conducted either *via* telephone or at the clinic. At the end of the follow-up, a total of 36 cases were lost to follow up, and 547 patients (93.8%) were followed to the end. The primary endpoint of the study was the occurrence of major adverse cardiac and cerebrovascular events (MACCEs), the composite of cardiac mortality, recurrence of myocardial infarction, cardiac shock, stroke, revascularization, or stent thrombosis. The secondary endpoint was all-cause mortality. Two experienced physicians adjudicated the endpoint events based on a review of medical records. Cardiac mortality was defined as death from any cardiac causes. Recurrence of myocardial infarction was defined as a novel myocardial infarction in the target vessel. Stent thrombosis was confirmed by an angiography. Cardiac shock was defined as a state of hypoperfusion resulting from a low cardiac output due to heart failure. Stroke was divided into fatal and non-fatal ischemic strokes. Revascularization was defined as revascularization involving either targeted or non-targeted vessels. A death from any cause was defined as an all-cause death. Bleeding events were defined as any bleeding event. Patients were censored at the last follow-up, on 24 December 2020, or whichever came first.

### Statistical analysis

The sample size was calculated with the following formula: $n\;=\;\frac{\left(\text{Zα}\sqrt{2\bar{pq}}\pm \text{Zβ }\sqrt{p}0q0+p1q1\right)2}{\left(p1-p0\right)}$

 Continuous variables are presented as ‘mean ± SD’ for normal distribution; otherwise, medians and interquartile ranges (IQRs) are used. Categorical variables are expressed as percentages. To analyze categorical variables, a chi-square (χ^2^) test was applied. Multiple group comparisons were performed using ANOVA. The Mann-Whitney *U* test or Kruskal–Wallis variance analysis was conducted for analyzing non-normal distribution. Multiple logistic regression analysis was employed to study the association of new permanent BBB with the severity of coronary atherosclerosis, as calculated by the Gensini scoring system. The Kaplan-Meier survival curve was employed to analyze the potential predictive efficacy of new permanent RBBB at baseline for the prognosis of new onset STEMI in patients with stent placement. SPSS 23 was used for the statistical analysis, with *p* < 0.05 indicating statistical significance.

## Results

### Baseline characteristics

A total of 547 patients with new onset STEMI who underwent PCI were retrospectively included in the study. The risk factors for CAD and baseline blood biochemical profiles of all patients were categorized into three group: no BBB, new onset permanent RBBB, or new onset permanent LBBB (Table 1). We found that patients with LBBB had higher levels of apolipoprotein B and low-density lipoprotein-cholesterol (LDL-C) than those with RBBB and no BBB (all *p* < 0.05). Patients with new LBBB and RBBB had higher triglyceride than those with no BBB (all *p* < 0.05).

**TABLE 1:** Baseline characteristics.

	All patients (*n* = 547)	No BBB (*n* = 467)	New permanent LBBB (*n* = 29)	New permanent RBBB (*n* = 51)	t/Z/χ^2^	*p*
Male n (%)	460 (84.1)	390 (83.5)	27 (93.1)	43 (84.3)	2.272	0.321
Age (years)	57.18 ± 11.84	57.10 ± 11.63	56.38 ± 12.35	58.29 ± 13.51	0.301	0.740
Hypertension	238 (43.5)	203 (43.5)	12 (41.4)	23 (45.1)	0.106	0.948
Diabetes mellitus	138 (25.2)	109 (23.3)	12 (41.4)a	17 (33.3)	6.169	0.046
Duration of diabetes mellitus (years)	8.86 ± 7.42	8.84 ± 7.71	8.05 ± 3.85	8.81 ± 6.20	0.059	0.943
DM treatment						
Diet only	9 (7.2)	7 (7.0)	2 (18.2)	0 (0.0)	3.565	0.407
Oral hypoglycemic drugs	57 (46.0)	47 (47.0)	3 (27.3)	7 (53.8)		
Insulin	58 (46.8)	46 (46.0)	6 (54.5)	6 (46.2)		
Smoking					4.848	0.254
Never smoker	227 (41.5)	202 (43.3)	8 (27.6)	17 (33.3)		
	8 (1.5)	7 (1.5)	0 (0.0)	1 (2.0)		
Former smoker	312 (57.0)	258 (55.2)	21 (72.4)	33 (64.7)		
						
Current smoker						
Alcohol drinking					1.038	0.904
Never drinking	331 (60.5)	285 (61.0)	16 (55.2)	30 (58.8)		
Former drinking	86 (15.7)	71 (15.2)	5 (17.2)	10 (19.6)		
Current drinking	130 (23.8)	111 (23.8)	8 (27.6)	11 (21.6)		
Family history of CAD n (%)	224 (410)	190 (40.7)	10 (34.5)	24 (47.1)	1.302	0.521
SBP (mmHg)	122.59 ± 19.97	123.32 ± 19.36	117.45 ± 21.49	118.82 ± 23.83	2.191	0.113
DBP (mmHg)	77.46 ± 13.15	77.57 ± 12.85	78.34 ± 15.82	75.94 ± 14.33	0.421	0.657
Heart rate (bpm)	81.95 ± 14.60	82.44 ± 14.82	77.55 ± 12.53	79.92 ± 13.25	2.082	0.126
BMI (kg/m^2^)	25.63 ± 11.42	25.87 ± 12.04	24.41 ± 5.32	23.86 ± 6.56	0.703	0.495
HDL-C (mmol/L)	0.98 ± 0.23	0.98 ± 0.24	0.99 ± 0.21	0.92 ± 0.19	1.543	0.250
LDL-C (mmol/L)	2.91 ± 0.90	2.94 ± 0.90	2.52 ± 0.85a	3.12 ± 0.74b	3.249	0.040
TC (mmol/L)	4.59 ± 1.25	4.56 ± 1.26	4.74 ± 1.27	4.95 ± 1.06	1.938	0.145
TG (mmol/L)	1.49 (0.97, 2.64)	1.47 (0.92, 2.60)	1.84 (1.37, 4.38)	1.81 (1.29, 2.58)	6.849	0.033
ApoA1 (g/L)	1.19 ± 0.26	1.9 ± 0.24	1.15 ± 0.23	1.22 ± 0.38	0.847	0.429
ApoB (g/L)	0.92 ± 0.29	0.92 ± 0.29	0.79 ± 0.23a	0.98 ± 0.26b	3.202	0.042
Lp (a) (g/L)	172.35 (101.25, 298.80)	173.95 (103.06, 299.97)	256.25 (128.92, 426.28)	142.92 (83.95, 239.47)	4.867	0.088
Creatinine (mmol/L)	77.48 ± 23.94	77.96 ± 24.53	74.29 ± 20.08	74.97 ± 20.36	0.629	0.533
BUN (mmol/L)	5.35 ± 1.92	5.36 ± 1.92	5.60 ± 1.59	5.17 ± 2.16	0.479	0.620
Uric acid (mmol/L)	324.68 ± 90.12	326.03 ± 86.27	309.76 ± 83.74	320.89 ± 123.28	0.494	0.611
LVEF (%)	57.58 ± 9.85	57.56 ± 9.75	56.41 ± 11.52	58.45 ± 9.90	0.403	0.669
LVEDD (mm)	51.74 ± 6.36	51.79 ± 6.39	52.59 ± 6.92	50.80 ± 5.75	0.824	0.439
Gensini score	72.48 ± 40.08	70.81 ± 39.52	81.41 ± 48.96	82.75 ± 38.3	2.818	0.061
UPLMT n (%)	45 (8.2)	34 (7.3)	3 (10.3)	8 (15.7)	3.770	0.152
LAD n (%)	481 (87.9)	410 (87.8)	26 (89.7)	45 (88.2)	0.097	0.952
LCX n (%)	320 (58.5)	277 (59.3)	13 (44.8)	30 (58.8)	2.363	0.307
RCA n (%)	396 (72.4)	341 (73.0)	19 (65.5)	36 (70.6)	0.861	0.650
Three-vessel coronary artery disease n (%)	228 (41.7)	204 (43.7)	10 (34.5)	24 (47.1)	1.229	0.541
Number of stent implantation	1.08 ± 0.31	1 (1, 1)	1 (1, 1)	1 (1, 1)	2.475	0.290
TIMI of target vessel					7.434	0.115
TIMI grade 1	28 (5.1)	21 (4.5)	4 (13.8)	3 (5.9)		
TIMI grade 2	44 (8.0)	34 (7.3)	5 (17.2)	5 (9.8)		
TIMI grade 3	475 (86.8)	412 (88.2)	20 (69.0)	43 (84.3)		

a *p* < 0.05, new permanent LBBB, group compared to no BBB, group;

b *p* < 0.05, new permanent RBBB, group compared to no BBB, group.

DM: diabetes mellitus; BMI: body mass index; SBP, systolic blood pressure; DBP, diastolic blood pressure; BUN, blood urea nitrogen; Cr, Creatinine; TC, total cholesterol; TG, triglyceride; HDL-C, High-density lipoprotein cholesterol; LDL-C, Low-density lipoprotein-cholesterol; Apo-AI, Apolipoprotein A1; Apo-B, Apolipoprotein B; Lp (a), Lipoprotein (a); UPLMT, unprotected left main trunk; LAD, left anterior descending artery; LCX, left circumflex artery; RCA, Right coronary artery; LVEF, left ventricular ejection fraction (%); LVEDD, left ventricular end diastolic diameter, thrombolysis in myocardial infarction (TIMI).

### Mortality rate and incidence of MACCEs

The incidence rates of clinical outcomes during the follow-up (43.93 ± 24.56 months) for patients with new-onset STEMI who underwent PCI based on no BBB, with new permanent RBBB or LBBB are shown in Table 2 (all *p* < 0.05). The results indicate that patients with new permanent RBBB were vulnerable to all-cause mortality, cardiac mortality, revascularization, stroke, and cardiac shock than those with LBBB and no BBB (all *p* < 0.05); those with LBBB had higher incidence of cardiogenic shock than those with no BBB (*p* < 0.05); and those with no BBB had lower incidence of MACCEs than those with new permanent LBBB and RBBB (*p* < 0.05).

**TABLE 2:** Incidence of adverse outcomes.

	No BBB (*n* = 467)	New permanent LBBB (*n* = 29)	New permanent RBBB (*n* = 51)	χ^2^	*p*
All-cause mortality, n (%)	6 (1.3)	1 (3.4)	13 (25.5)^ab^	40.830	<0.001
MACCEs, n (%)	26 (5.6)	5 (17.2)^a^	21 (41.2)^ab^	47.146	<0.001
Cardiac death, n (%)	6 (1.3)	0 (0.0)	9 (17.6)^ab^	25.175	<0.001
Recurrence of myocardial infarction, n (%)	1 (0.2)	1 (3.4)	0 (0.0)	5.214	0.112
Stent thrombosis, n (%)	1 (0.2)	0 (0.0)	0 (0.0)	0.317	1.000
Revascularization, n (%)	14 (3.0)	1 (3.4)	6 (11.8)^ab^	6.675	0.036
Cardiac shock, n (%)	20 (4.3)	3 (10.3)	9 (17.6)^a^	11.791	<0.001
Stroke, n (%)	0 (0.0)	0 (0.0)	2 (3.9)^ab^	9.940	0.011
Bleeding events, n (%)	7 (1.5)	0 (0.0)	1 (2.0)	0.450	0.720

### Severity of coronary lesions

The baseline characteristics of participants according to the GS tertiles (first GS tertile <48, *n* = 182; second GS tertile: 49–84, *n* = 183; and third GS tertile ≥85, *n* = 182) are presented in Table 3. Age, high-density lipoprotein cholesterol, apolipoprotein AI, prevalence of diabetes mellitus, and hypertension were significantly different among the three GS tertiles (all *p* < 0.05). However, new permanent RBBB, new onset LBBB, and no BBB indicated no statistical differences with respect to GS based on the observed CAG findings (*p* > 0.05). Multivariate logistic analyses demonstrated that age (OR: 1.015, *p* = 0.033), high-density lipoprotein cholesterol (OR: 0.354, *p* = 0.003), apolipoprotein AI (OR: 0.481, *p* = 0.023), the prevalence of diabetes mellitus (OR: 1.499, *p* = 0.031), and incidence of hypertension (OR: 1.528, *p* = 0.009) were independently associated with the severity of coronary lesions in new onset STEMI patients (Table 4).

**TABLE 3:** Baseline characteristics of participants according to the Gensini score tertiles.

	1st tertile	2nd tertile	3rd tertile	t/Z/χ^2^	*p*
	≤48 (*n* = 182)	49–84 (*n* = 183)	≥85 (*n* = 182)		
Male (%)	160 (87.9)	154 (84.2)	146 (80.2)	4.026	0.134
Age (years)	55.40 ± 11.82	57.27 ± 11.36	58.86 ± 12.14^a^	3.953	0.020
Hypertension (%)	63 (34.6)	82 (44.8)	93 (51.1)^a^	10.248	0.006
Diabetes mellitus (%)	35 (19.2)	45 (24.6)	58 (31.9)^a^	7.764	0.021
Duration of diabetes mellitus (years)	7.43 ± 6.60	10.85 ± 8.99	8.51 ± 6.30	2.137	0.122
Diabetes mellitus treatment				0.892	0.926
Diet only	3 (7.1)	3 (9.1)	3 (6.1)		
Oral hypoglycemic drugs	20 (47.6)	13 (39.4)	24 (49.0)		
Insulin	19 (45.2)	17 (51.5)	22 (44.9)		
Smoking				2.885	0.577
Never smoker	70 (38.5)	79 (43.2)	78 (42.9)		
Former smoker	4 (2.2)	3 (1.6)	1 (0.5)		
Current smoker	108 (59.3)	101 (55.2)	103 (56.6)		
Alcohol drinking				0.698	0.952
Never drinking	114 (62.6)	110 (60.1)	107 (58.8)		
Former drinking	28 (15.4)	28 (15.3)	30 (16.5)		
Current drinking	40 (22.0)	45 (24.6)	45 (24.7)		
Family history of CAD	63 (34.6)	77 (42.1)	84 (46.2)	5.154	0.076
SBP (mmHg)	122.18 ± 19.82	123.17 ± 17.97	122.42 ± 22.01	0.121	0.886
DBP (mmHg)	76.52 ± 13.63	78.26 ± 12.00	77.59 ± 13.75	0.808	0.446
Heart rate	80.69 ± 13.91	81.76 ± 14.74	83.4 ± 15.09	1.586	0.206
BMI (kg/m^2^)	24.82 ± 6.39	25.34 ± 4.33	26.80 ± 18.45	1.297	0.274
HDL-C (mmol/L)	1.02 ± 0.27	0.96 ± 0.21a	0.94 ± 0.21^b^	6.223	0.002
LDL-C (mmol/L)	2.85 ± 0.86	2.92 ± 0.76	3.05 ± 1.01	1.793	0.168
TC (mmol/L)	4.61 ± 1.33	4.53 ± 0.96	4.70 ± 1.41	0.638	0.529
TG (mmol/L)	1.48 (0.94, 2.90)	1.43 (0.87, 2.63)	1.80 (1.12, 2.53)	1.919	0.383
ApoA1 (g/L)	1.23 ± 0.31	1.19 ± 0.24	1.14 ± 0.21^a^	4.368	0.013
ApoB (g/L)	0.90 ± 0.25	0.90 ± 0.24	0.96 ± 0.36	2.171	0.115
Lp(a) (g/L)	165.45 (93.61, 293.82)	176.70 (94.96,317.80)	183.8 (108.35,288.44)	0.497	0.780
Cr (mmol/L)	79.56 ± 23.16	75.32 ± 22.45	77.61 ± 26.00	1.423	0.242
BUN (mmol/L)	5.35 ± 2.02	5.28 ± 1.90	5.43 ± 1.86	0.303	0.739
Uric acid (μmol/L)	323.13 ± 78.68	325.30 ± 92.66	325.60 ± 98.21	0.040	0.961
LVEF (%)	57.21 ± 10.01	57.89 ± 9.76	57.65 ± 9.82	0.224	0.799
LVEDD (mm)	52.43 ± 6.61	51.79 ± 5.86	51.00 ± 6.54	2.333	0.098
Electrocardiography diagnosis				8.154	0.086
No BBB	160 (87.9)	161 (88.0)	146 (80.2)		
New permanent RBBB	10 (5.5)	9 (4.9)	10 (5.5)		
New permanent LBBB	12 (6.6)	13 (7.1)	26 (14.3)		

a *p* < 0.05 compared to the first tertile;

b *p* < 0.05 compared to the second tertile.

**TABLE 4:** Independent risk factors for severity of coronary lesions evaluated by Gensini score.

Variables	B	Se	Wald	*p*	OR	95% CI	
						Lower limit	Upper limit
Age	0.015	0.007	4.547	0.033	1.015	1.001	1.028
Hypertension	0.424	0.163	6.731	0.009	1.528	1.110	2.106
Diabetes mellitus	0.405	0.187	4.678	0.031	1.499	1.039	2.164
HDL-C	−1.039	0.353	8.667	0.003	0.354	0.178	0.707
ApoA1	−0.731	0.322	5.146	0.023	0.481	0.256	0.906

### Clinical outcomes

**FIGURE 2:**
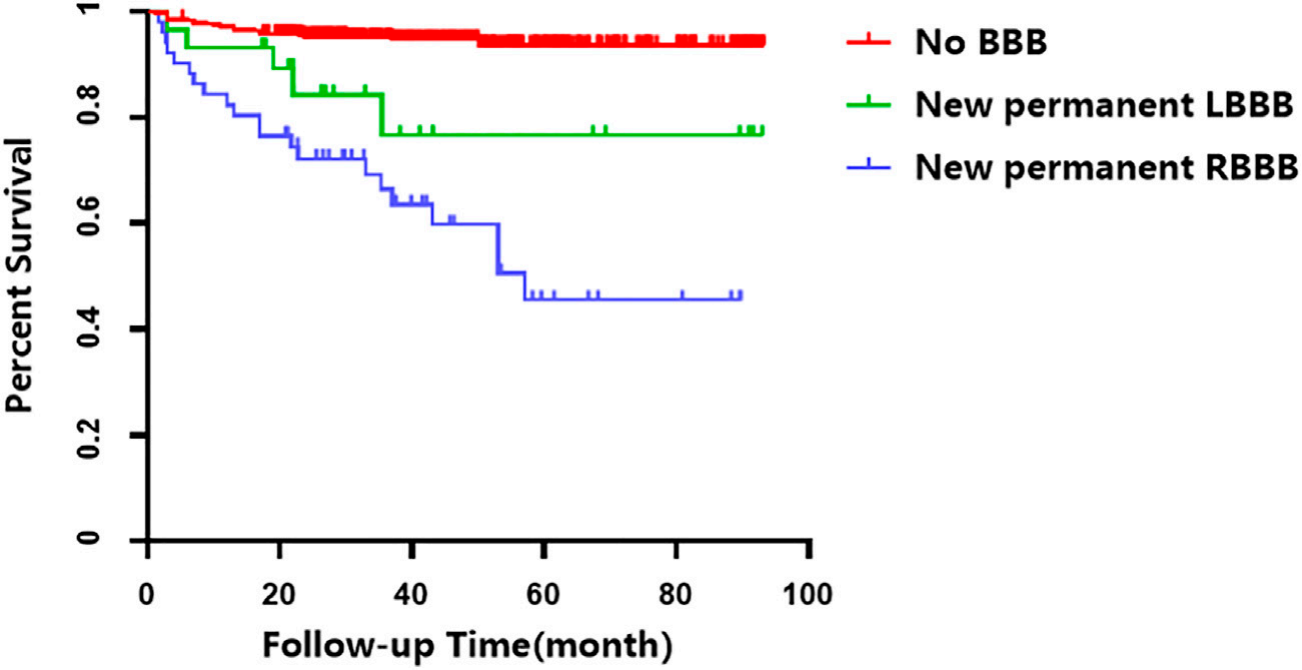
Cumulative survival of MACCEs in patients with STEMI who underwent PCI.

**FIGURE 3:**
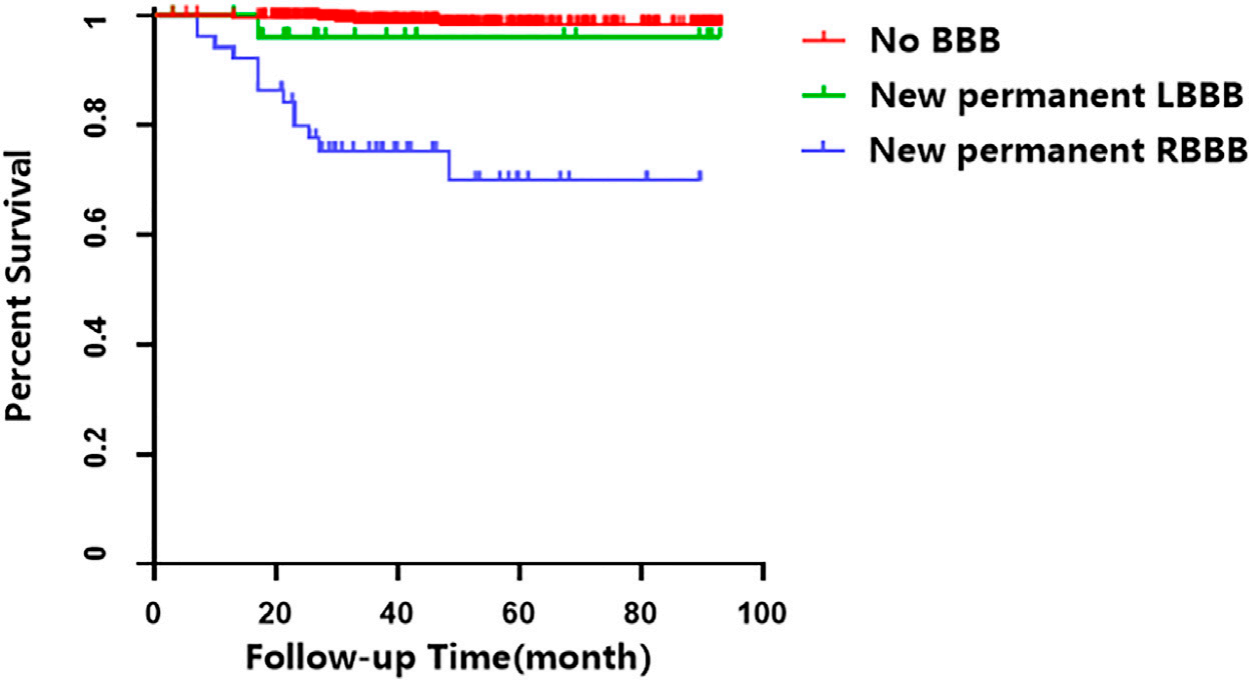
Cumulative survival analysis of all-cause mortality in patients with STEMI who underwent PCI.

**FIGURE 4:**
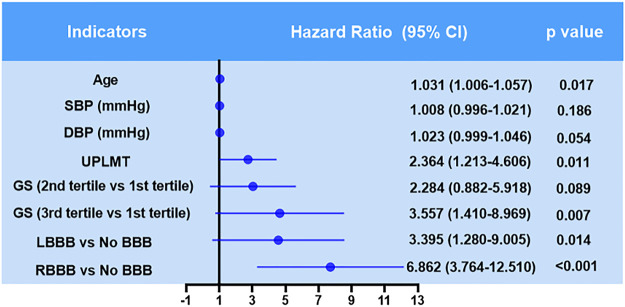
Hazard ratios for MACCEs in patients with new onset STEMI who underwent PCI.

**FIGURE 5:**
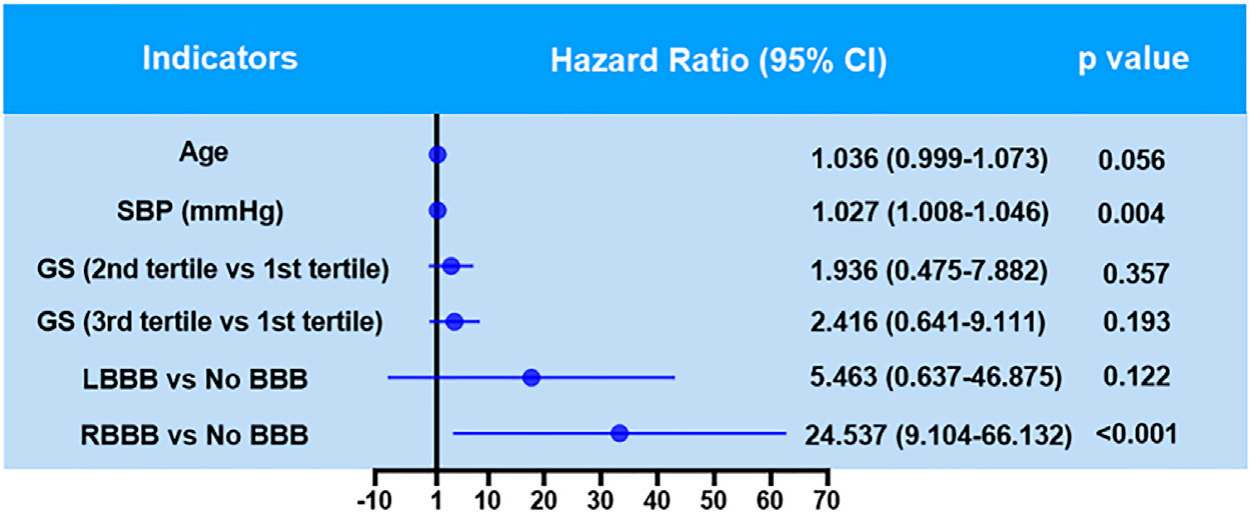
Hazard ratios for all-cause mortality in patients with new onset STEMI who underwent PCI.

The Kaplan-Meier analysis showed that the incidence of MACCEs differed significantly among groups of patients with no BBB, with new onset permanent RBBB and LBBB, and with new onset STEMI who underwent PCI (χ^2^ = 80.231 *p* < 0.001, Figure 2). Pairwise comparisons further indicated that patients with new permanent RBBB had a higher incidence of MACCEs than those with new permanent LBBB (χ^2^ = 5.107, *p* = 0.021, Figure 2). Likewise, patients with new permanent RBBB had a higher incidence of MACCEs than those with no BBB (χ^2^ = 81.253, *p* < 0.001, Figure 2). Patients with new permanent LBBB had a higher risk of developing future MACCEs than those with no BBB (χ^2^ = 7.783, *p* = 0.005, Figure 2). Moreover, there was a significant difference in all-cause mortality among those with no BBB and with new onset permanent RBBB and LBBB groups (χ^2^ = 86.558, *p* < 0.001, Figure 3). Furthermore, pairwise comparisons demonstrated that patients with new permanent RBBB had a higher incidence of all-cause mortality than those with new onset permanent LBBB (χ^2^ = 4.131, *p* = 0.042, Figure 3). Patients with new onset permanent RBBB had a higher risk of future all-cause mortality than those with no BBB (χ^2^ = 87.812, *p* < 0.001, Figure 3). Univariate Cox regression analysis indicated that age, systolic and diastolic blood pressure, higher GS, unprotected left main coronary artery (UPLMCA) lesions, RBBB, and LBBB were all potential predictors of MACCEs (Table 5, all *p* < 0.05). Subsequent multivariate analysis showed that new permanent RBBB and LBBB were independently correlated with a high risk of MACCEs and adjusted HRs of 6.862 (95% CI, 3.764–12.510) and 3.395 (95% CI, 1.280–9.005), respectively, compared to patients with no BBB (both *p* < 0.05, Table 5; Figure 4). Age (HR: 1.031, 95% CI: 1.006 to 1.057, *p* = 0.017), UPLMCA (HR: 2.364, 95% CI: 1.213 to 4.606, *p* = 0.011), and increase of the GS (third tertile vs. First tertile, HR: 3.557, 95% CI: 1.410 to 8.969, *p* = 0.007) were all independent risk factors for MACCEs in new onset STEMI patients post-PCI (Table 5; Figure 4). Univariate Cox regression analysis indicated that age, systolic blood pressure, higher GS, RBBB, and LBBB were all potential predictors of MACCEs (Table 6, all *p* < 0.05). The results of a subsequent multivariate analysis demonstrated that new permanent RBBB was independently correlated with an increased risk of all-cause mortality with an adjusted HRs of 24.537 (95% CI: 9.104–66.132), compared to patients with no BBB (*p* < 0.05, Table 6). Likelihood estimates revealed that systolic pressure (HR:1.027, 95% CI: 1.008 to 1.046, *p* = 0.004) was an independent predictor of all-cause mortality in new onset STEMI patients post-PCI (Table 6; Figure 5).

**TABLE 5:** Potential predictors for the incidence of MACCEs in patients with new onset STEMI who underwent PCI.

Variables	Univariate			Multivariate		
	HR	95% CI	*p*	HR	95% CI	*p*
Sex (Male/Female)	1.077	0.525–2.210	0.840			
Age	1.041	1.017–1.066	0.001	1.031	1.006–1.057	0.017
Hypertension	1.005	0.581–1.737	0.985			
Diabetes mellitus	1.458	0.817–2.603	0.202			
Smoking						
Former smoker vs. Never smoker	1.417	0.190–10.589	0.734			
Current smoker vs. Never smoker	1.216	0.689–2.145	0.500			
Alcohol drinking						
Former smoker vs. Never drinking	1.322	0.620–2.822	0.470			
Current smoker vs. Never drinking	1.644	0.892–3.031	0.111			
Family history of CAD	0.806	0.455–1.427	0.459			
SBP (mmHg)	1.014	1.001–1.028	0.040	1.008	0.996–1.021	0.186
DBP (mmHg)	1.025	1.005–1.045	0.014	1.023	0.999–1.046	0.054
Heart rate	1.003	0.985–1.023	0.727			
BMI (kg/m^2^)	0.964	0.922–1.009	0.114			
HDL-C (mmol/L)	0.587	0.175–1.964	0.387			
LDL-C (mmol/L)	1.070	0.773–1.482	0.684			
TC (mmol/L)	1.093	0.869–1.374	0.446			
TG (mmol/L)	0.994	0.856–1.155	0.938			
ApoA1 (g/L)	0.306	0.089–1.053	0.060			
ApoB (g/L)	1.746	0.714–4.267	0.222			
Lp(a) (g/L)	0.999	0.997–1.001	0.266			
Creatinine (mmol/L)	0.999	0.989–1.012	0.939			
BUN (mmol/L)	0.974	0.841–1.129	0.728			
Uric acid (μmol/L)	0.999	0.997–1.003	0.936			
EF (%)	0.999	0.972–1.027	0.947			
LVEDD (mm)	0.971	0.926–1.017	0.210			
UPLMT	5.336	2.922–9.743	<0.001	2.364	1.213–4.606	0.011
LAD	1.749	0.630–4.850	0.283			
LCX	1.581	0.885–2.824	0.122			
RCA	1.904	0.928–3.906	0.079			
Three-vessel coronary artery disease	1.672	0.969–2.887	0.065			
Number of stent implantation	1.061	0.461–2.440	0.889			
TIMI of target vessel						
TIMI grade 2 vs. TIMI grade 1	0.725	0.221–2.378	0.595			
TIMI grade 3 vs. TIMI grade 2	0.479	0.189–1.213	0.121			
2nd tertile vs. 1st tertile	2.674	1.037–6.892	0.042	2.284	0.882–5.918	0.089
3rd tertile vs. 1st tertile	6.007	2.503–14.419	<0.001	3.557	1.410–8.969	0.007
LBBB vs. No BBB	3.697	1.417–9.648	0.008	3.395	1.280–9.005	0.014
RBBB vs. No BBB	8.922	5.015–15.873	<0.001	6.862	3.764–12.510	<0.001

**TABLE 6:** Potential predictors for the incidence of all-cause mortality in patients with new onset STEMI who underwent PCI.

Variables	Univariate			Multivariate		
	HR	95% CI	*p*	HR	95% CI	*p*
Sex (Male/Female)	2.211	0.849–5.755	0.104			
Age	1.047	1.007–1.088	0.021	1.036	0.999–1.073	0.056
Hypertension	0.533	0.205–1.388	0.198			
Diabetes mellitus	2.051	0.838–5.018	0.116			
Smoking						
Former smoker vs. Never smoker	1.029	0.578–1.744	0.980			
Current smoker vs. Never smoker	1.082	0.442–2.648	0.862			
Alcohol drinking						
Former smoker vs. Never drinking	1.963	0.671–5.744	0.218			
Current smoker vs. Never drinking	1.237	0.423–3.620	0.698			
Family history of CAD	1.431	0.596–3.439	0.423			
SBP (mmHg)	1.021	1.001–1.042	0.048	1.027	1.008–1.046	0.004
DBP (mmHg)	1.030	0.999–1.062	0.061			
Heart rate	0.996	0.966–1.028	0.823			
BMI (kg/m^2^)	0.970	0.888–1.060	0.501			
HDL-C (mmol/L)	0.791	0.120–5.221	0.808			
LDL-C (mmol/L)	1.045	0.632–1.730	0.863			
TC (mmol/L)	1.126	0.798–1.589	0.501			
TG (mmol/L)	1.068	0.895–1.274	0.467			
ApoA1 (g/L)	1.155	0.221–6.033	0.864			
ApoB (g/L)	1.533	0.376–6.248	0.551			
Lp(a) (g/L)	0.997	0.994–1.001	0.141			
Creatinine (mmol/L)	0.999	0.981–1.018	0.911			
BUN (mmol/L)	0.976	0.771–1.236	0.842			
Uric acid (μmol/L)	1.001	0.996–1.006	0.769			
EF (%)	0.999	0.955–1.046	0.990			
LVEDD (mm)	0.920	0.841–1.006	0.067			
UPLMT	2.292	0.671–7.825	0.186			
LAD	1.262	0.293–5.442	0.755			
LCX	1.710	0.657–4.451	0.272			
RCA	2.221	0.651–7.579	0.203			
Coronary artery three-ressel disease	2.053	0.839–5.025	0.115			
Number of stent implantation	0.567	0.087–3.686	0.553			
0000FF						
TIMI of target vessel						
TIMI grade 2 vs. TIMI grade 1	1.112	0.101–12.288	0.931			
TIMI grade 3 vs. TIMI grade 2	0.930	0.124–6.987	0.943			
Gensini group						
2nd tertile vs. 1st tertile	2.106	0.527–8.420	0.292	1.936	0.475–7.882	0.357
3rd tertile vs. 1st tertile	4.013	1.119–14.395	0.033	2.416	0.641–9.111	0.193
LBBB vs. No BBB	3.834	0.460–31.953	0.214	5.463	0.637–46.875	0.122
RBBB vs. No BBB	23.491	8.907–61.954	<0.001	24.537	9.104–66.132	<0.001

## Discussion

In this retrospective cohort study, we have drawn the following conclusions: 1) the occurrence of new permanent RBBB and LBBB is an independent predictor for MACCEs in patients with new onset STEMI post PCI; 2) patients with new permanent RBBB are most likely at higher risk for future all-cause mortality and MACCEs than those with new permanent LBBB and with no BBB; 3) having no BBB with new permanent RBBB or LBBB is not associated with coronary artery lesions as evaluated by the GS; and 4) new permanent RBBB in patients with new onset STEMI who underwent PCI had a worse prognosis than those with LBBB or no BBB.

Previous studies suggested that new onset BBB might indicate a larger territory of AMI involving the proximal branches of the cardiac conduction system or a delayed conduction caused by the severely damaged ventricular myocardium. Either indicator might have concealed the ST-segment elevation (Willems et al., 1985). Current guidelines state that symptomatic patients with new or presumably new LBBB should be treated as STEMI equivalents, which usually is correlated with worse clinical characteristics and prognosis (Neeland Ian et al., 2012). Our data are consistent with the guidelines that emphasize the importance of new LBBB in clinic practice, particularly in patients with successful revascularization (Borja et al., 2018). However, the predictive efficacy of RBBB in ACS remains to be determined (Antman Elliott et al., 2004; Mozid Abdul et al., 2015; Chan et al., 2016; Alkindi et al., 2020; Paul et al., 2020). The inconsistency among previous studies might be caused by the different criteria for patient enrollment and study endpoints. In a previous study, 202,268 primary care patients with no-known significant cardiovascular disease were followed-up for 7.8 years. A previous work found that RBBB was a predictor for pacemaker implantation, a risk factor associated with increased incidence of heart failure in both sexes, and weakly associated with cardiac death in men (Juntao et al., 2018). In a prospective study of 17,437 patients with AMI, results revealed that, although isolated BBB was directly correlated with high-risk clinical characteristics such as three-vessel and left main disease, it could not be used solely to predict increased hospital mortality in AMI patients (Paul et al., 2020). In contrast, in a recent study of 50,974 patients with acute cardiac events, RBBB was found to be independently and significantly correlated with hospital mortality (Mozid Abdul et al., 2015). Likewise, multiple studies have indicated that RBBB is an independent predictor of in-hospital short- and long-term mortality in the context of patients with different types of ACS presentations, including unstable angina and myocardial infarction (McCullough Peter et al., 2005; Widimsky et al., 2012; Chan William et al., 2016; Peter Vibe et al., 2019). Importantly, it is now becoming accepted that RBBB, as a predictor of poor outcomes for ischemic cardiomyopathy, also serves as an independent indicator for the risk of decreased right ventricular ejection fraction (Melgarejo-Moreno et al., 2015). However, it is imperative to optimize the risk stratification in myocardial infarction patients post-PCI, especially for the identification of easily neglected and potential prognostic factors (Sabe Marwa et al., 2016; Yi et al., 2020). Moreover, previous studies failed to address the association between RBBB and prognosis post-PCI.

In our retrospective study, we found no correlations between new permanent LBBB and RBBB and the severity of coronary artery atherosclerosis assessed by GS, which is consistent with prior studies (Wang et al., 2022). Moreover, compared to patients with no BBB, patients with new permanent RBBB were associated with an increased overall risk of MACCEs and all-cause mortality in new onset STEMI patients who underwent PCI, even after adjusting for comorbidities after a long-term follow-up. Therefore, our overall findings are similar to those from the previous reports that new onset RBBB was a high-risk signal for new onset STEMI (Prakriti et al., 2020; Zhu et al., 2022). We also demonstrated that patients with new permanent RBBB were more prone to adverse events, including MACCEs and all-cause mortality compared to those with new permanent LBBB and with no BBB. One possible pathophysiological mechanism for our discovery is that RBBB is more susceptible to dys-synchronized ventricular contractions, resulting in progressive decreased ventricular filling, than LBBB, which in turn leads to an increased risk of developing MACCEs (Figueroa-Triana et al., 2021). Thus, compared to patients with LBBB, this might explain why a new permanent RBBB patient seems more susceptible to ischemic cardiomyopathy (Miller et al., 2015) and at a higher risk for MACCEs and all-cause mortality. In addition, we found that patients with BBB were more likely to have higher levels of triglyceride, while patients with LBBB were more likely to have higher levels of LDL-C and apolipoprotein B. Therefore, patients with BBB had more concomitant risk factors than patients with no BBB, leading to a riskier MACCEs profile. However, the exact underlying mechanisms have yet to be fully understood. In our study, the average duration of follow-up with patients was longer than that in other studies, which might have provided additional prognostic indication of new permanent RBBB in new onset STEMI patients who underwent PCI. Moreover, RBBB is considerably influenced by some non-cardiac factors that may interfere with the predictive value of RBBB (Bussink et al., 2013; Sorensen et al., 2013). Yet, this does not seem to be the case in our study since only new onset STEMI patients with new permanent RBBB or LBBB were included. Based on previous studies and our experience in clinical practice, we found that cardiac ischemic symptoms and the appearance of a new permanent RBBB, especially in the absence of ST-segment elevation, were rather common (Peter Vibe et al., 2019). It is important to mention that these occurrences could be easily misunderstood and overlooked, leading to failed attempts to perform an urgent coronary angiography and, often, disastrous consequences.

### Study limitations

First, our work was a retrospective observational study with a relatively small sample size that might have caused selection bias, especially for new onset LBBB and RBBB patients, which could overestimate the predictive value of RBBB and its outcome. Therefore, our findings need to be validated in future prospective and multicenter studies. Second, considering that our study was observational, confounding factors could influence our results, and new onset BBB might have been misclassified in some patients. Although patients did not have any known history of CAD or new onset BBB, our study was not structured to provide insights into the pathophysiology underlying the differences in MACCEs seen with RBBB.

## Conclusion

Our study suggests that the occurrence of new permanent RBBB in patients with new onset STEMI may serve as a significant warning sign and be applied as a useful risk stratifier for a new high-risk onset STEMI in patients who underwent PCI.

## Data Availability

The original contributions presented in the study are included in the article/Supplementary Material, further inquiries can be directed to the corresponding authors.
